# Impact of point-of-care ultrasound on the hospital length of stay for internal medicine inpatients with cardiopulmonary diagnosis at admission: study protocol of a randomized controlled trial—the IMFCU-1 (Internal Medicine Focused Clinical Ultrasound) study

**DOI:** 10.1186/s13063-019-4003-2

**Published:** 2020-01-08

**Authors:** Ximena Cid, David Canty, Alistair Royse, Andrea B. Maier, Douglas Johnson, Doa El-Ansary, Sandy Clarke-Errey, Timothy Fazio, Colin Royse

**Affiliations:** 1 Department of Surgery, University of Melbourne, Melbourne, VIC Australia; 2 Department of Medicine and Community Care, Royal Melbourne Hospital, Parkville, VIC Australia; 3 Department of Anesthesia and Perioperative Medicine, Monash Health, Melbourne, VIC Australia; 4 Department of Anesthesia and Pain Management, Royal Melbourne Hospital, Parkville, VIC Australia; 5 Department of Surgery, Royal Melbourne Hospital, Parkville, VIC Australia; 6 Department of Medicine and Aged Care, @AgeMelbourne, The Royal Melbourne Hospital, University of Melbourne, Melbourne, VIC Australia; 7 Department of Human Movement Sciences, @AgeAmsterdam, Amsterdam Movement Sciences, Vrije Universitet, Amsterdam, The Netherlands; 8 Department of Health Professions, Swinburne University of Technology, Melbourne, VIC Australia; 9 Statistical Consulting Centre, University of Melbourne, Parkville, VIC Australia; 10 Business Intelligence Unit, Melbourne Health, Parkville, VIC Australia; 11 Department of Medicine and Radiology, Melbourne Medical School, University of Melbourne, Parkville, VIC Australia; 12 Outcomes Consortium, Cleveland Clinic, Cleveland, OH USA

**Keywords:** Randomized controlled trial, Echocardiography, Focused assessment sonography, Lung ultrasound, Internal medicine

## Abstract

**Background:**

Point-of-care ultrasound (POCUS) is emerging as a reliable and valid clinical tool that impacts diagnosis and clinical decision-making as well as timely intervention for optimal patient management. This makes its utility in patients admitted to internal medicine wards attractive. However, there is still an evidence gap in all the medical setting of how its use affects clinical variables such as length of stay, morbidity, and mortality.

**Methods/design:**

A prospective randomized controlled trial assessing the effect of a surface POCUS of the heart, lungs, and femoral and popliteal veins performed by an internal medicine physician during the first 24 h of patient admission to the unit with a presumptive cardiopulmonary diagnosis. The University of Melbourne iHeartScan, iLungScan, and two-point venous compression protocols are followed to identify left and right ventricular function, significant valvular heart disease, pericardial and pleural effusion, consolidation, pulmonary edema, pneumothorax, and proximal deep venous thrombosis. Patient management is not commanded by the protocol and is at the discretion of the treating team. A total of 250 patients will be recruited at one tertiary hospital. Participants are randomized to receive POCUS or no POCUS. The primary outcome measured will be hospital length of stay. Secondary outcomes include the change in diagnosis and management, 30-day hospital readmission, and healthcare costs.

**Discussion:**

This study will evaluate the clinical impact of multi-organ POCUS in internal medicine patients admitted with cardiopulmonary diagnosis on the hospital length of stay. Recruitment of participants commenced in September 2018 and is estimated to be completed by March 2020.

**Trial registration:**

Australian and New Zealand Clinical Trial Registry, ACTRN12618001442291. Registered on 28 August 2018.

## Introduction

Patients admitted to internal medicine wards with cardiorespiratory symptoms can be difficult to assess and treat as they are usually older, have multiple co-morbidities, and take multiple medication. Traditionally, internal medicine physicians rely on the medical history and physical examination, collectively known as the clinical evaluation, to derive a differential diagnosis and formulate an initial management plan. However, it has been reported that clinical evaluation alone is frequently inaccurate in determining the correct diagnosis [1–4]. The delay of making a precise diagnosis and starting an appropriate management could be detrimental for patient outcome. Further management is refined by investigations including medical imaging to confirm or rule out the differential diagnosis. However, unnecessary investigations can be associated with high cost and patient risk such as radiation exposure, contrast-induced nephropathy, and transfer of acutely ill patients to an investigation laboratory.

Ultrasonography has been used in medicine for at least 50 years, is non-invasive and without ionizing radiation. Only in the last two decades have ultrasound machines evolved to produce portable, low-cost units that are readily available for use at the bedside, facilitating its clinical uptake. The terms “clinical ultrasound” and “point-of-care ultrasound” (POCUS) are used to describe a bedside ultrasound examination performed by the treating doctor, as an adjunct to clinical evaluation [5]. Its use has become very common in some medical specialties such as emergency medicine, anesthesia, and critical care. Several prior studies have demonstrated validity and reliability [6–14]. The organ scanned depends on the clinical question. By detecting omitted abnormalities in the physical examination [1, 2, 4] and improving the hemodynamic status evaluation [15, 16], heart POCUS has become a useful tool in the evaluation of undifferentiated shock, guiding resuscitation, or as part of the preoperative evaluation in patients undergoing surgery [17–21] Furthermore, lung ultrasound has proved to be superior than physical examination and chest X-ray diagnosing pneumonia, interstitial syndrome (including pulmonary edema), and pleural effusion [22, 23]. In the emergency department and critical care setting, it is now frequently used in the approach of patients with dyspnea, in which lung POCUS alone or in combination with heart POCUS has demonstrated to be very precise distinguishing the primary cause [24–34].

Studies quantifying clinical impact of POCUS have shown that its use led to change diagnosis and modify management plans in 30%–80% of the cases depending on the clinical scenario [17, 18, 35–38]. Most of these studies have investigated imaging of one particular organ [17, 35, 39–42]. However, a multi-organ approach may better align with the initial assessment of complex cases in internal medicine as they are frequently multi-organ in presentation [5, 43]. This is especially true among cardiopulmonary patients in which a broad range of differential diagnoses can be proposed [44]. In these patients, a combined heart and lung POCUS can identify the cause of dyspnea in most of the cases or significantly narrow the range of diagnoses [26, 33, 34]. In addition to heart and lung POCUS, lower extremity vein POCUS can be used to accurately identify proximal deep venous thrombosis (DVT) [12, 45], which might cause pulmonary embolus and be the cause of shortness of breath or cardiovascular collapse. A multi-organ POCUS of the heart, lungs, and lower extremity veins has already been tested in a randomized trial with respiratory patients from the emergency department, reporting superiority of POCUS to standard diagnostic tests alone for establishing a correct diagnosis within 4 h [27].

We expect that the addition of a heart, lung, femoral, and popliteal vein POCUS in cardiopulmonary patients admitted to internal medicine wards will have a positive impact in the timely diagnosis formulation and impact on decision-making. Moreover, it is plausible that improving diagnosis and altering management plans may lead to improvement in the workflow and reduction of the length of hospital stay.

### Objectives and hypothesis

The primary aim of the study is to determine whether a heart, lung, and lower extremity vein POCUS reduces the length of hospital stay of patients admitted to internal medicine wards with a cardiopulmonary diagnosis by > 24 h.

The secondary aims are to evaluate the impact of POCUS on: (1) change in diagnosis and management plan; (2) 30-day hospital readmission; and (3) in-hospital health costs.

### Trial design

The IMFCU-1 trial is a single-center, prospective, randomized, parallel group, unblinded, superiority trial with 1:1 allocation ratio. The intervention is a bedside ultrasound examination, which makes blinding not feasible.

## Methods

Ethics approval for the study was obtained from Melbourne Health Human Research Committee on 27 June 2018 (protocol reference 2018.200). The study has been conducted in accordance with the Declaration of Helsinki and registered with the Australian and New Zealand Clinical Trial Registry on 28 August 2018 (ACTRN12618001442291). Table 1 in shows all the items of the World Health Organization trial registration dataset.

**Table 1:** World Health Organization trial registration dataset for IMFCU-1

Data category	Information
Primary registry and trial identifying number	Australian and New Zealand Trial Registration, ACTRN12618001442291
Date of registration	28 August 2018
Prospective registration	Yes
Primary sponsor	Royal Melbourne Hospital
Public title	A bedside ultrasound in general medicine patients with cardiopulmonary diagnosis
Scientific title	A randomized trial of focused cardiac, lung, and femoral and vein ultrasound on the length of stay in internal medicine admissions with a cardiopulmonary diagnosis. IMFCU-1 study.
Date of first enrolment	3 September 2018
Target sample size	250
Recruiting status	Recruiting (103 recruited)
URL	U111112185271
Study type	Interventional
Study design	Randomized controlled trial parallel
Phase	Not applicable
Country of recruitment	Australia
Contacts	Prof Colin Royse (principal investigator) Address: Level 6, Centre of Medical Research, Royal Parade, Parkville, VIC 3052, Australia. Telephone: (61)383445673 Email: colin.royse@heartweb.com Affiliation: Department of Surgery, University of Melbourne Department of Anesthesia and Pain Management, Royal Melbourne Hospital
Key inclusion & exclusion criteria	Inclusion criteria: Adult patients (aged 18 years or older) admitted to general medicine unit at the Royal Melbourne Hospital with a cardiopulmonary diagnosis, expected to remain in hospital longer than 24 h. Exclusion criteria: Already admitted longer than 24 h Admitted for social reasons rather than medical Have received an echocardiography within four weeks before admission or a CT chest scan during the admission process before enrolment
Health conditions or problems studied	Heart failure, asthma, COPD, pneumonia, PE, unspecified dyspnea
Intervention	A bedside ultrasound done by a physician trained in POCUS. The ultrasound takes around 20 min to be performed. The quality of the report will be assessed by a second expert who will check the images and videos recorded.
Primary outcome	LOS at the hospital
Secondary outcome	Incidence of new diagnosis and Incidence of changing management. These two outcomes will be assessed only in the interventional group. The treating physician will be asked to fill in a form with the initial diagnosis and plan of management. This form is a checklist describing further investigations (blood test and imaging), consultation to another specialist and medication prescribed (diuretics, antibiotics, etc.). After performing and revealing to them the findings of the bedside ultrasound, the treating physician will be asked to fill a second form that is exactly the same than the first one. The difference between both will be analyzed as “change of management” due to our intervention. Health costs: this outcome will be assessed by the sum of the following three components: (1) cost per day at the hospital; (2) cost of the pathology investigation; (3) cost of the imaging tests.

*COPD* chronic obstructive pulmonary disease, *IMFCU* internal medicine focused clinical ultrasound, *LOS* length of stay, *PE* pulmonary embolism, *POCUS* point-of-care ultrasound

Methods are reported in accordance with the Guidance for protocols of clinical trials (SPIRIT) [46]. The SPIRIT checklist is shown in Additional file 1.

### Study setting

The trial is performed at the Royal Melbourne Hospital (RMH), a tertiary, public, university-affiliated teaching hospital, with 706 beds located in Victoria, Australia. Participants are recruited from the internal medicine wards, which are logistically divided into long-stay and short-stay units containing around 68 and 32 beds, respectively. Approximately 30% of the internal medicine patients are hospitalized due to cardiopulmonary conditions.

### Eligibility criteria

Patients admitted to the internal medicine ward with a preliminary cardiopulmonary diagnosis are invited to participate in the study. Eligible participants are selected every workday morning by internal medicine physicians during their handover. After presenting the new cases, physicians are asked to identify the cardiopulmonary cases. For the purpose of the study, a cardiopulmonary diagnosis has been defined as a medical suspicion that the main health problem of the patient is related to one of the following heart or lung conditions: heart failure; acute coronary syndrome; pulmonary embolism (PE); pneumonia; decompensated chronic pulmonary obstructive disease; asthmatic crisis; cardiogenic syncope; interstitial pulmonary disease; cardiac valve disease; pleural effusion; or pericardial effusion.

#### Inclusion criteria

- Age ≥ 18 years;
- Less than 24 h since admission to the internal medicine ward;
- Cardiopulmonary diagnosis defined by an internal medicine specialist.

#### Exclusion criteria

- Previous echocardiography during the four weeks before hospital admission;
- Computed tomography chest during the current hospital admission;
- Requiring infectious disease isolation (contact, drops, or respiratory precaution);
- Unable to consent (by themselves or a third person who is nominated/identified as their next of kin).

### Intervention

The intervention is a POCUS performed by an internal medicine physician with previous experience in POCUS and the certification of iHeartScan, iLungScan, and Focused Cardiac Ultrasound courses from the Educational Ultrasound Group of the University of Melbourne (XC).

POCUS is performed with an X-Porte portable ultrasonography machine (Sonosite, Bothwell, Andover, MA, USA) using a 1–5-MHz transthoracic and 6–13-MHz linear ultrasound probes. The ultrasound is performed at the patient’s bedside, taking an average of 20 min to be completed.

**Fig. 1:**
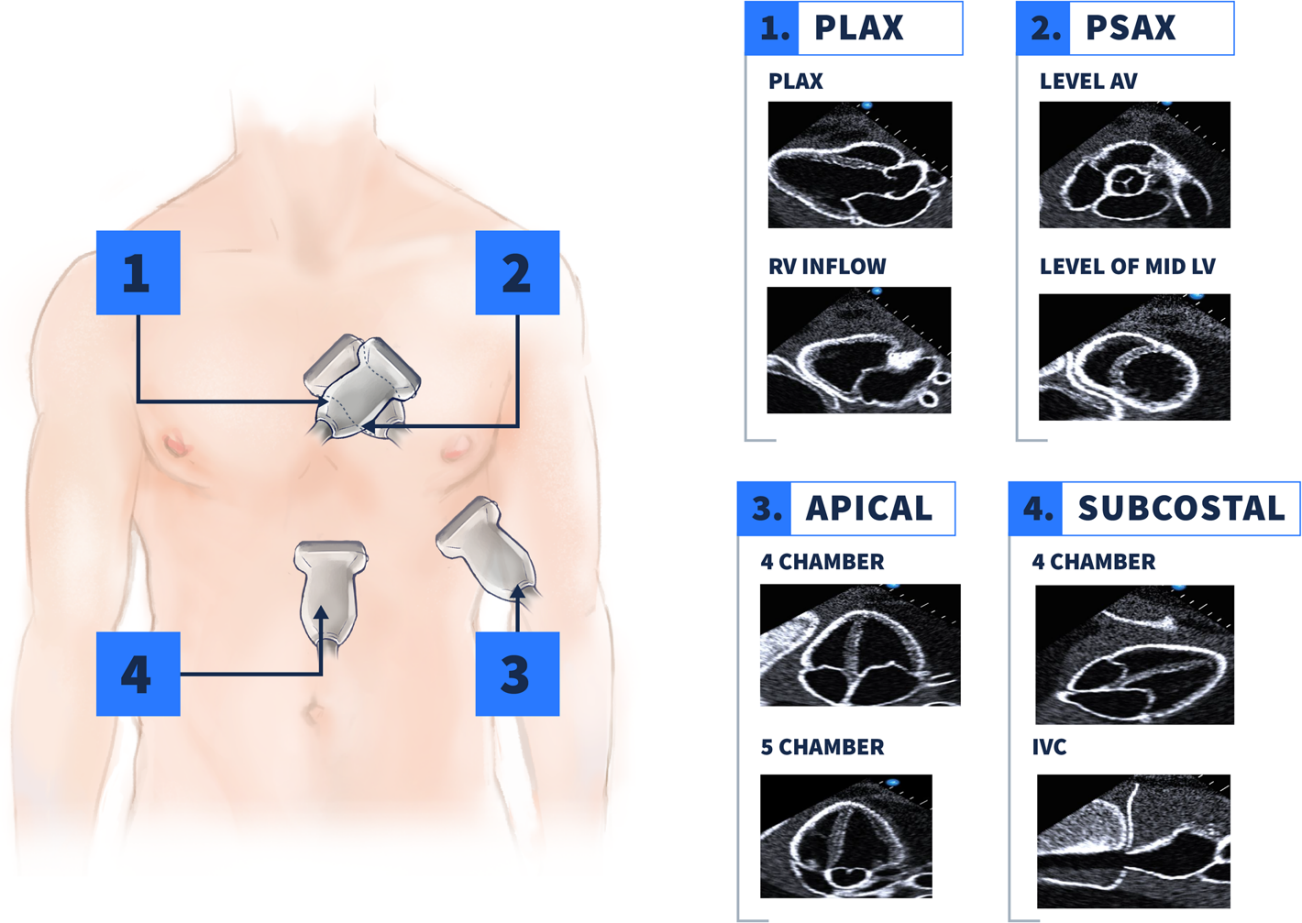
Ultrasonography windows assessed in heart POCUS. Four anatomical windows are used to assess eight views of the heart: (1) At the level of the fourth intercostal space lateral to the left border of the sternum, PLAX and RV inflow are recorded. (2) The second window is technically the same than the first, from PLAX the probe is rotated in clock direction ending in the PSAX. Two views are recorded at this point, one at the level of the aortic valve and other at the level of papillary muscle or mid left ventricle. (3) Apical window is found about the fifth intercostal space between the mid clavicular line ant the anterior axillary line. In this window the views assessed are A4C and apical five-chamber. (4) Subcostal window involves two views: subcostal four-chamber view of the heart and the IVC view where the IVC can be identified ending in the right atrium. A4C, apical four chambers, IVC inferior vena cava, PLAX parasternal long axis, POCUS point-of-care ultrasound, PSAX parasternal short axis, RV right ventricle

Assessment of the heart and lungs is performed based on the iHeartScan and iLungScan protocols designed and validated by the Ultrasound Education Group of the University of Melbourne [47–49]. Heart structure and function are assessed using two-dimensional (2D) images and color flow Doppler; spectral Doppler is not included in this study to facilitate timely completion of the ultrasound and to increase its reproducibility. Heart POCUS involves four anatomical windows to record eight views (Fig. 1): parasternal long axis; right ventricle (RV) inflow; parasternal short axis at the level of the aortic valve; parasternal short axis at the level of the papillary muscle; apical four chambers (A4C); apical five chambers; subcostal four chambers; and subcostal inferior vena cava.

The following variables are assessed and reported: volume and systolic function of the LV and RV; left atrial filling pressure based on the interatrial septum movement; significant regurgitation or stenosis of the valves; presence or absence of pericardial effusion; and diameter and collapsibility of the inferior vena cava. Definitions for each variable abnormality are summarized in Table 2. A final statement about the hemodynamic condition will be written as follows: normal; hypovolemia; vasodilated; primary systolic dysfunction; primary diastolic dysfunction; systolic and diastolic dysfunction; and/or RV dysfunction as described by Royse et al. [15] and summarized in Table 3.

**Table 2:** Variables assessed and definitions of abnormality findings in heart POCUS

	Variable assessed	Definitions
LV volume	LVEDD	Normal LVEDD 3–5.6 cm LV dilated > 5.6 cm Hypovolemia < 3 cm
LV systolic function	Overall subjective impression	Normal – Reduced – Increased
	Difference between diameters in diastole and systole (LVEDD–LVESD) in PLAX view	Normal 28–44 mm Reduced < 28 mm Increased > 44 mm
	Difference between areas in diastole and systole (LVEDA–LVESA) in PSAX view	Normal 50–65 mm^2^ Reduced < 50 mm^2^ Increased > 65 mm^2^
RV size	Compared to LV size	Normal < 2/3 of LV size
	RVEDD	Normal < 4 cm Increased > 4 cm
RV systolic function	Overall subjective impression	Normal – Decreased
LA size	LA diameter in PLAX or A4C views	Normal < 3.5 cm
	LA area in A4C view	Normal < 20 cm^2^ Increased > 20 cm^2^
LA filling pressure	Inter-atrium septum movement	Normal: systolic reversal of the inter-atrium septum High filling pressure: fixed curvature of the inter-atrium septum to the right Low filling pressure: systolic buckling of the inter-atrium septum
Cardiac valves	Leaflets appearance and thickness Opening of the valve Presence of reverse jet	Significant aortic stenosis: An opening < 1.5 cm in PLAX or Heavy calcification with inability to see the valve opening
		Significant aortic regurgitation: A jet that runs on the wall of the LV outflow track A jet that is wider than 25% of the diameter of LVOT A jet that extends down to the ventricle > 2.5 cm
		Significant mitral stenosis: Impaired opening of the mitral valve A hockey stick appearance of one or both of the mitral leaflets
		Significant mitral regurgitation: Regurgitation jet covering > 20% of the LA area in A4C or PLAX A turbulent jet that runs along the wall of the atrium Prominent flail mitral valve leaflet or rupture papillary muscle
		Significant tricuspid regurgitation: Any edge-tracking jet Any central jet > 5 cm^2^
Pericardial effusion	Presence of anechoic space between parietal and visceral pericardium	Significant pericardial effusion is defined as > 0.5 cm in any view
Inferior vena cava	Diameter of the inferior vena cava in the subcostal view during normal breathing	Maximum diameter in cm and percentage of collapsibility during normal inspiration are reported. Estimation of the right atrium pressure is informed as follows: IVC < 2.1 cm collapsing > 50% ➔ RAP: 3 mmHg IVC > 2.1 cm collapsing < 50% ➔ RAP: 15 mmHg Values between the two above ➔ RAP:8 mmHg

*A4C* apical four chambers, *LA* left atrium, *LV* left ventricle, *LVEDA* left ventricle end-diastole area, *LVEDD* left ventricle end-diastole diameter, *LVESA* left ventricle end-systole area, *LVESD* left ventricle end-systole diameter, *PLAX* parasternal long axis, *POCUS* point-of-care ultrasound, *PSAX* parasternal short axis, *RAP* right atrium pressure, *RVEDD* right ventricle end-diastole diameter

**Table 3:** Hemodynamic state definitions

	Normal	Hypovolemia	Vasodilated	Primary systolic failure	Primary diastolic failure	Systolic and diastolic failure	RV failure^a^
LV volume	Normal	Decreased	Normal	Increased	Normal/decreased	Increased	RV increased
LV systolic function	Normal	Normal/Decreased	Increased	Decreased	Normal	Decreased	RV decreased
LA filling pressure	Normal	Decreased	Normal	Normal	Increased	Increased	Increased

Hemodynamic state is defined based on LV volume, LV systolic function, and LA filling pressure

^a^ RV failure can be a hemodynamic state by itself or in combination with LV failure

*LA* left atrium, *LV* left ventricle, *RV* right ventricle

**Fig. 2:**
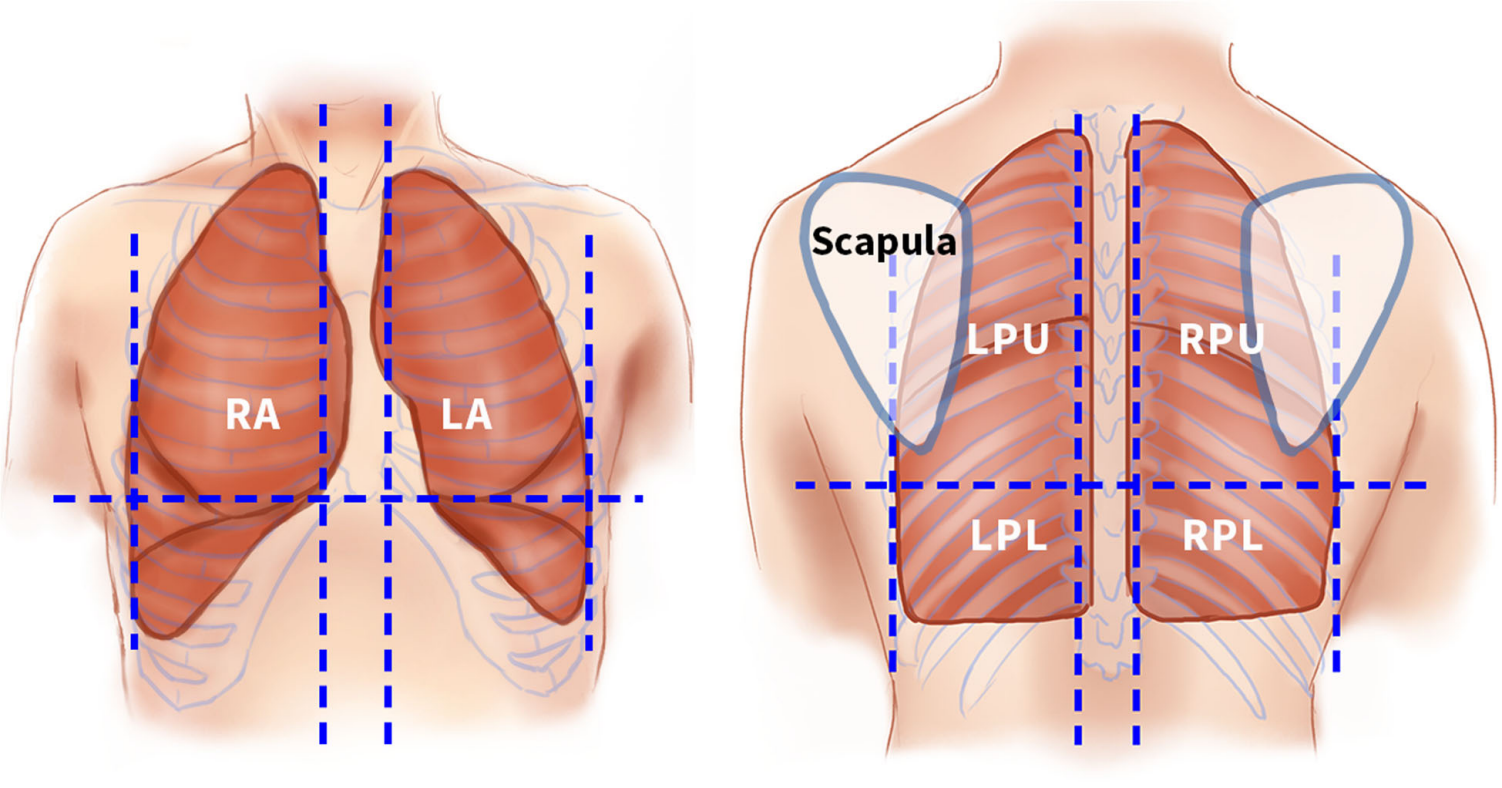
Anatomical zones scanned in lung POCUS. Illustrations of the front (*left*) and back (*right*) of the chest showing the six anatomical zones scanned. LA left anterior, LPL left posterior lower, LPU left posterior upper, POCUS point-of-care ultrasound, RA right anterior, RPL right posterior lower, RPU right posterior upper

The lungs are scanned by division into three anatomical zones as previously reported by Ford et al. [49] (Fig. 2). The anterior zone goes from the sternum edge to the mid-axillary line posteriorly; the upper posterior zone is defined by the mid-axillary line anteriorly, the spinous processes of the thoracic spine posteriorly, and the inferior tip of the scapular inferiorly: and the lower posterior zone is defined by the mid-axillary line anteriorly, the spinous process of the thoracic spine posteriorly, and the inferior rip of the scapula superiorly. Abnormal findings are recorded as: collapse; consolidation; alveolar/interstitial syndrome; pneumothorax; and/or pleural effusion. Definitions are described in Table 4. Normal lung pattern is defined as the presence of normal lung sliding, reverberation artefacts from the pleural, and absence of any of the pathologies described.

**Table 4:** Definitions of ultrasound lung abnormalities

Abnormal lung patterns	Definition / ultrasound findings
Alveolar/Interstitial syndrome	3 or more B-lines in a single rib space B-lines were defined as hyperechoic, vertical artifacts arising from the pleural line and reaching the bottom of the screen without fading
Collapse or atelectasis	Loss of lung volume, increased tissue density, and hyperechoic static air bronchograms
Consolidation	Tissue-like pattern or “hepatization” with minimal volume loss and the presence of dynamic air bronchograms
Pneumothorax	Absence of lung sliding and lung pulse
Pleural effusion	Anechoic space between the parietal and visceral pleura with movement with the respiratory cycle. Significant pleural effusion is defined as > 1 cm. An estimation of the volume of a pleural effusion in milliliters (ml) will be done multiplying by 200 the distance in cm in the vertical plane from the diaphragm to the inferior lung border at the junction of the collapsed lung and aerated lung

**Fig. 3:**
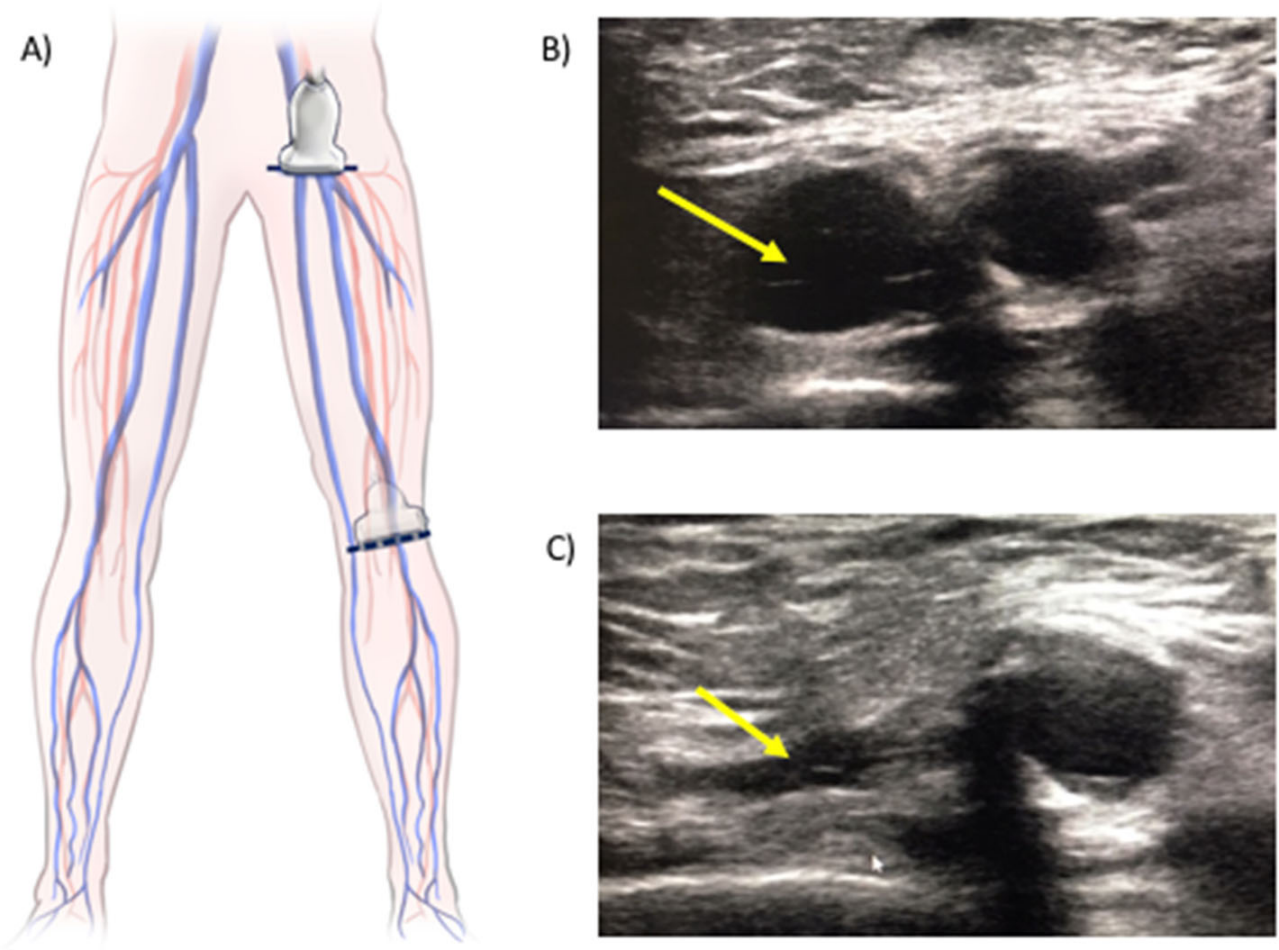
Femoral and popliteal vein POCUS. **a** The *illustration* shows the two points of the lower extremities assessed for DVT: the common femoral vein at the groin level and popliteal vein at the popliteal fossa. **b**, **c** Ultrasound images showing the vein marked with yellow arrows before (**b**) and after (**c**) external compression has been applied. In this case, the vein is entirely collapsible, consistent with absence of a DVT. DVT deep venous thrombosis, POCUS point-of-care ultrasound

Femoral and popliteal veins are assessed for intravascular thrombosis using the two-point compression technique [12, 42] (Fig. 3), in which the vein collapsibility is evaluated in two points for each lower extremity: the common femoral vein at the level of the groin and the popliteal vein in the popliteal fossa. A DVT is defined as the inability to completely collapse the vein with the ultrasound probe. This technique has proved a sensitivity of 96.1% and specificity of 96.6% diagnosing proximal DVT when it has been compared to standard vein ultrasound performed by radiologists [12, 42].

**Fig. 4:**
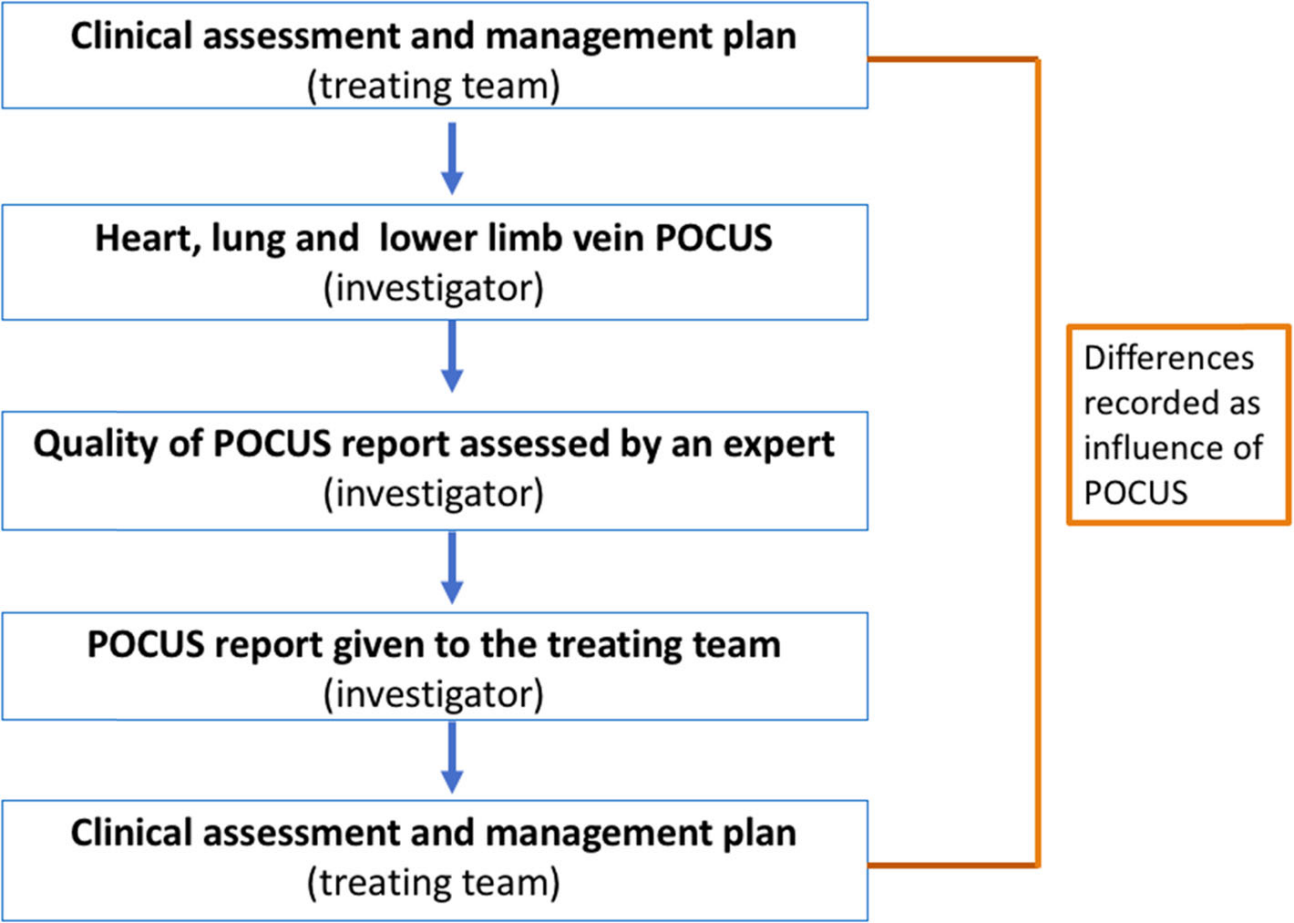
Steps involved in the intervention group. In the intervention group, a POCUS of the heart, lungs, and femoral and popliteal veins is performed at the patient’s bedside. The report summarizing the main findings is assessed by a second expert in POCUS before it is given to the treating team. The treating team is requested to fill out forms about their clinical assessment and management plan before and after receiving the POCUS report. The difference between forms will be recorded as influence of POCUS. POCUS point-of-care ultrasound

Once the test has been performed, a structured report summarizing the main findings is written. The quality of this report is immediately assessed by a second POCUS expert reviewing the images recorded. There are three experts participating in this study as quality evaluators (CR, AR, and DV), all of them with at least 10 years of experience in POCUS. The revised report is given to the treating team without any direction of management, who in turn are requested to fill out forms about their clinical assessment before and after receiving the POCUS report (Fig. 4).

The intervention will not be performed or will be stopped after being already started if the patient refers intolerable discomfort during the procedure or in any clinical condition that involves urgent management such as cardiorespiratory arrest, pain, or respiratory distress. In these cases, if some of the variables were already assessed, a report with partial information will be given to the treating team.

The control group follows the standard care pathway, which does not include POCUS. Diagnosis and management will be based on clinical evaluation and other investigations. Ultrasound examinations are not precluded such as those performed by cardiology or radiology staff, but POCUS of the heart, lungs, or lower extremity veins are not allowed during the time that the participant remains admitted to an internal medicine ward.

There are no restrictions in medication use or further standard investigations in any of the two groups.

### Outcomes

#### Primary outcome

The primary outcome is the difference in the median of length of hospital stay between the intervention group and the control group. Length of stay (LOS) is defined as number of hours from admission to the internal medicine ward to hospital discharge.

#### Secondary outcomes

Impact on diagnosis and management will be reported as follows: (1) number and proportion of patients in whom a new diagnosis was found with POCUS; (2) number and proportion of patients in whom the main cardiorespiratory diagnosis was changed after POCUS; (3) number and proportion of patients who had a management modification. Management includes adding or removing medications to treat cardiorespiratory conditions (e.g. diuretics), requesting further investigations, and consulting to another specialist.

Readmission to the hospital will be presented as proportion of patients readmitted to the hospital during the next 30 days after hospital discharge in both groups.

Healthcare costs involve total costs spent in each patient during their hospital stay presented in Australian dollars. The data will be organized in several categories (bed stay, imaging tests, pathology investigations). The average cost of each category will be compared between groups.

### Participant timeline

**Fig. 5:**
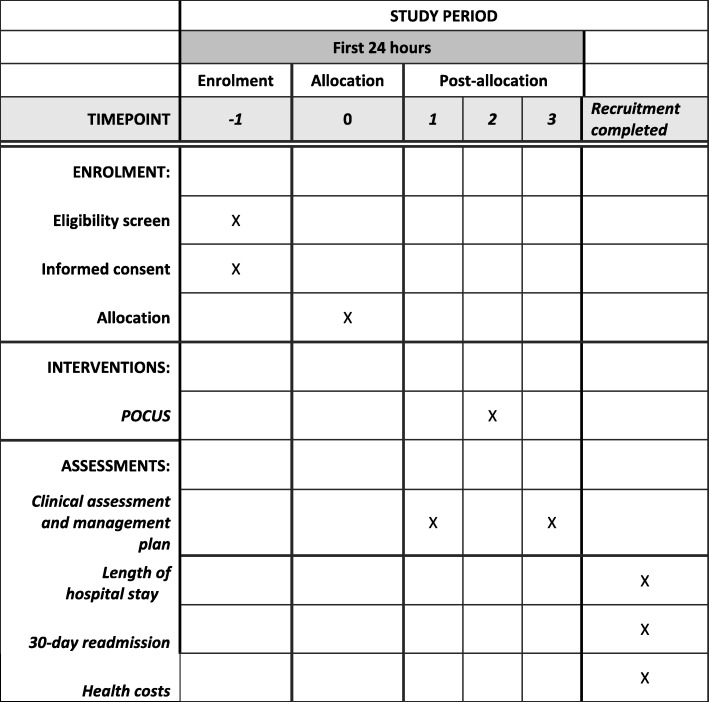
Schedule of enrolment, intervention, and assessments

Screening for eligibility, enrolment, allocation, and intervention is performed on the same day (Fig. 5). No follow-up of participants is done after hospital discharge. Data about LOS, 30-day readmission, and costs will be obtained from the hospital electronic databases after finishing the recruitment.

### Sample size

A sample size of 122 participants in each group has been estimated, which is being rounded up to a total of 250 participants to allow withdrawals. This estimation has been calculated using the statistical software G Power Version 3, based on a t-test of log transformed LOS in hours from internal medicine wards of the RMH (median 103 h), a clinically important effect on LOS defined as ≥ 24 h, power of 80%, and alpha of 0.05.

### Recruitment

One of the investigators (XC) attends every internal medicine handover from Monday to Friday, ensuring that all the new cardiopulmonary patients from the previous night shift are screened for eligibility. Once the internal medicine physicians have identified the potential participants, the order in which these patients will be approached is done following a randomized sequence created by computer software. In this way, selection bias is significantly reduced. Participants received verbally information and a written document about what it means to participate in this study and how the study is conducted. Once they have agreed to participate, they are asked to sign an informed consent form. In case participants cannot give their consent due to cognitive impairment, a person responsible or a person already stablished as their medical treatment decision maker will be asked to sign the consent on their behalf.

**Fig. 6:**
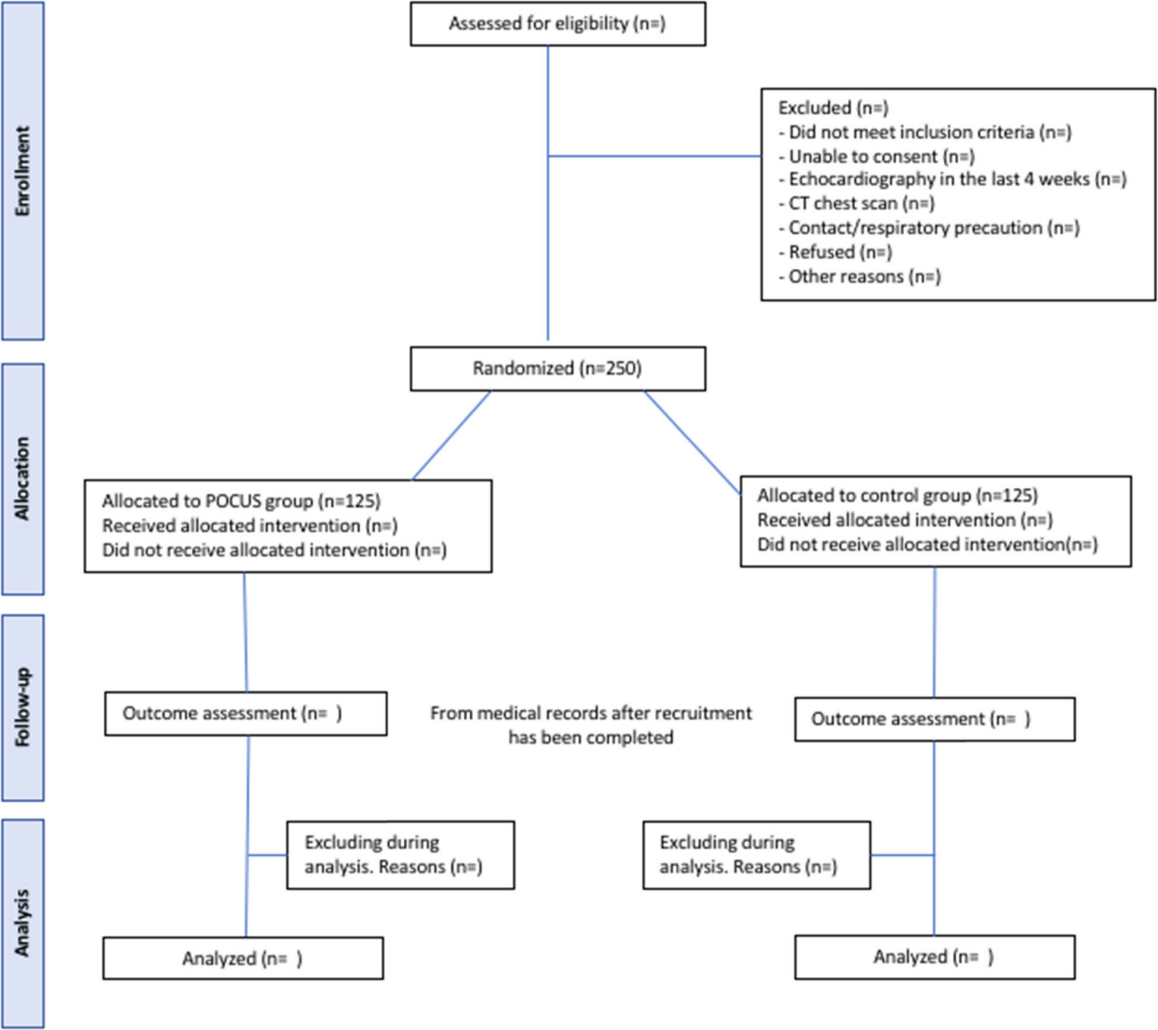
Proposed Consolidated Standard of Reporting Trials (CONSORT) *flow chart* for IMFCU-1 study

The recruitment rate for the past eight months has been 15 participants per month. Therefore, we expect to complete the recruitment in eight months. A proposed of Consolidated Standard of Reporting Trials (CONSORT) flowchart is shown in Fig. 6.

### Allocation

Participants are randomly assigned to the intervention or control group with a 1:1 ratio. The allocation sequence is based on permuted blocks of random size, generated by computer software. Blocks sizes are 4, 6, or 8. Sealed, numbered, double-layered, opaque envelopes are used for concealment. The concealment was performed by a non-investigator. Enrolment and allocation will be done by the same investigator after participant has signed the informed consent form. The only person who has access to the allocation sequence is the main investigator, who is not involved in the recruitment process.

### Blinding

Due to the nature of the intervention, blinding was not possible. It was considered unethical to perform POCUS and conceal the results in the control group. However, in order to reduce detection bias, the person assigning the primary outcome will be blinded to what group each participant belongs.

### Withdrawal from the study

If participants withdraw from the study, their data will not be available for analysis. To date this has not occurred and we anticipate a very low withdrawal number.

### Data collection methods

Demographic data and baseline information will be gathered prospectively by one of the investigators from the medical notes. Baseline data is detailed in Table 5.

**Table 5:** Patient basal data to be collected

Demographic data	Age (years)
	Gender (female)
	Height (cm)
	Weight (kg)
	BMI (kg/m^2^)
Prior medical conditions	Hypertension
	Congestive cardiac failure
	Angina
	Myocardial infarction
	Coronary intervention
	Known significant valve disease
	Valve replacement
	Cardiac arrhythmia
	Pulmonary hypertension
	COPD
	Interstitial lung disease
	Asthma
	Smoking
	Diabetes
	Known renal failure
	Stroke
	HIV
	Venous thromboembolism
	Cancer (type, active/remission, metastasis)
	Chronic liver disease
	Hypothyroidism
	Hyperthyroidism
	Cognitive impairment/dementia
Chronic medication	Antihypertensive
	Beta-blockers
	Antiplatelet
	Anticoagulant
	Systemic steroids
	Diuretics
	Chemotherapy
	Other
Cardiopulmonary symptoms	Dyspnea/shortness of breath
	Chest pain
	Palpitations
	Cough
	Fever suspected to be respiratory or cardiac
	Lower limb edema
	Altered state of consciousness
	Other
Vital signs	Blood pressure (mmHg)
	Heart rate (beats per minute)
	Temperature (°C)
	Respiratory rate (breaths per minute)
	O_2_ saturation (%)

*BMI* body mass index, *COPD* chronic obstructive pulmonary disease, *HIV* human immunodeficiency virus

#### Primary outcome

Hospital LOS is obtained from the hospital operating system and is not influenced by any of the investigators. A list with full name, patient number, and the date of admission, but blinded to allocation, will be sent to the hospital Business Intelligence Unit to generate the LOS data.

If patients are transferred from physical internal medicine wards to the “Hospital in the Home” (HITH) program, an acute general care program in the patients homes, the LOS will be added to the LOS on internal medicine wards.

#### Secondary outcomes

New diagnosis and change in management will be evaluated using forms about clinical assessment completed by the treating physician before and after the findings of POCUS are revealed to them. Both forms are exactly the same. The difference between them will be interpreted as the effect of the intervention. (Fig. 4).

The information requested in these forms includes (Additional file 2):

- The hemodynamic state of the patients from the following options: normal; hypovolemia; primary diastolic failure; primary systolic failure; systolic and diastolic failure; vasodilation; and RV failure.
- Describing physical examination findings specifying LV function, significant valve regurgitation or valve stenosis, pericardial effusion, suspicion of PE, abnormalities of the lungs, and evidence of DVT.
- Recording the most likely diagnosis.
- Detailing further investigations. In this section they will have a list of pathology tests, imaging tests, and consultations to other medical specialties. They will be asked to mark all the further investigations that they are requesting.
- Describing the type of treatment prescribed from five options: (1) heart failure treatment (defined by one of the following treatment: diuretics, vasodilators, and/or fluid restriction); (2) COPD/asthma treatment (defined as the use of bronchodilators and/or systemic steroids); (3) antibiotics; (4) anticoagulation in therapeutic dose; and (5) “other” in case it is a different treatment from the four above. More than one option can be chosen.

The seniority of doctors who will complete the forms is restricted to internal medicine specialists and specialist trainees.

Readmission to the hospital data will be gathered from hospital operating system in the same form that has been explained for the primary outcome. Planned readmissions will be excluded, analyzing only unplanned readmissions in the following 30 days after hospital discharge.

Information about economic health will be gathered directly from the Business Intelligence Unit. This unit centralizes all the information related to health costs and analyses it for administrative purposes.

Once the trial has finished recruiting, we will send them the blinded list of patients specifying the admission and discharge date. Hospital costs include total costs and categories such as bed stay, imaging tests, and pathology tests.

#### Harms

Performing POCUS is highly unlikely to cause unintended effects. It is a non-invasive and ionizing radiation-free imaging test with no severe side effects reported other than infrequent patient discomfort [50]. However, severe bruising from probe manipulation over the skin and patient falls during the procedure set-up (e.g. when a patient is transferred from the chair to their bed) will be documented and reported in the final results as total number of events. If any of these events occur, management will have to refer the event to the treating physician.

### Statistical methods

The primary outcome, LOS in hours, will be analyzed using Student’s t-test on the log scale, in anticipation of skewness in raw LOS. Log transforming might normalize a skewed data, making outliers unlikely. However, a cut-off of 30 days will be applied to LOS to avoid the effect of extremely long hospital stays. Statistical analysis will be done using the software SPPS (Statistical Package for the Social Sciences), version 26.

For patients who die in hospital, death will be treated as hospital discharge for the primary analysis as the unpublished mortality rate of patients admitted to internal medicine wards of the RMH is low (2.7%). However, a sensitivity analysis will be conducted to explore the impact of this approach. Missing data for the primary outcome are not expected as it is anticipated that LOS will be available for every patient in the study. If for some reason this information is missing, those patients will not be included in the primary statistical analysis.

For the primary outcome, significance is defined as *p* < 0.05. Secondary outcomes will be analyzed using parametric or non-parametric tests according to the type of data, whether the data are skewed, and whether repeated measures are used. As the secondary endpoints involve more pairwise comparisons, significance will be defined as *p* < 0.01 to reduce the risk of Type 1 error from multiple pairwise comparisons. All estimates will be reported with 95% confidence intervals. All analysis will be based on the principle of intention-to-treat.

### Data management

All data will be entered electronically using numerical codes. Paper records will be stored in files in a locked filing cabinet, in a locked room in the Department of Surgery of the University of Melbourne. Electronic data are stored in password-protected databases, available only to researchers involved in the study. The primary outcome and health cost data are generated from the hospital electronic systems and not under the influence of investigators. Other data will undergo double data entry range checks for data value errors.

Due to the small trial size, there is neither Data Monitoring Committee (DMC) established nor stopping rules applied. There is no planned interim analysis.

The final results of this study are intended to be disseminated through publications in peer-reviewed medical journals. After publishing, data about demographic, primary, and secondary outcomes will be shared to other researchers who request it to the principal investigator with a project proposal and with acceptance of release of data by the Melbourne Health Human Research Ethics Committee.

## Discussion

This study will show whether the addition of a multi-organ POCUS in internal medicine patients reduces the LOS at the hospital. LOS was selected as the main outcome as it was considered objective, reliable data and clinically relevant to both the patient and the healthcare system. Demonstrating an impact in LOS will encourage physicians around the world to incorporate this technique in their routinely practice.

The novelty of this study is that it is the first randomized trial assessing the impact on LOS of a multi-organ focused ultrasound in internal medicine. Last year, Mozzini et al. [51] described a positive impact of repetitive lung ultrasounds on the LOS in patients with heart failure admitted to the internal medicine ward. Based on their results, we are optimistic about finding a positive effect this time assessing a multi-organ focused ultrasound which not only evaluate lung but also heart and DVT.

The limitations of the study are that the outcomes are short-term and related to hospital admission. This study will provide clinical data that can serve to assess feasibility and sample size for a trial investigating morbidity and mortality outcomes. A randomized pilot study investigating focused cardiac ultrasound in patients undergoing fractured neck of femur surgery showed a group separation of 30-day mortality and morbidity outcomes of 39% favoring the use of cardiac ultrasound [52] and a lower 12-month mortality [53]. The sample size of this study, however, is too small to investigate morbidity and mortality outcomes for the cardiopulmonary admissions to internal medicine wards. Further, the use of ultrasound in not a medical intervention but rather an investigation. The behavior change consequent on the information is the actual mechanism whereby improved outcomes can occur. If the treating medical staff choose to ignore the findings, or not act appropriately upon them, then the value of the ultrasound examination is diminished. This problem is most likely to occur at the start of the study where skepticism regarding the POCUS study exists and reduces over time as the additional knowledge form a feedback loop on the clinician’s diagnostic approach.

## Trial status

Recruiting.

Recruitment began on 3 September 2018.

## Supplementary information

**Additional file 1.** SPIRIT 2013 Checklist: Recommended items to address in a clinical trial protocol and related documents.

**Additional file 2.** Clinical Assessment.

## Abbreviations

A4C: Apical four chambers
COPD: Chronic obstructive pulmonary disease
DVT: Deep venous thrombosis
LA: Left atrium
LOS: Length of stay
LV: Left ventricle
LVEDA: Left ventricle end-diastole area
LVEDD: Left ventricle end-diastole diameter
LVESA: Left ventricle end-systole area
LVESD: Left ventricle end-systole diameter
PE: Pulmonary embolism
PLAX: Parasternal long axis
POCUS: Point-of-care ultrasound
PSAX: Parasternal short axis
RMH: Royal Melbourne Hospital
RV: Right ventricle
RVEDD: Right ventricle end-diastole diameter
