# Supplementary material for: Impact of point-of-care ultrasound on the hospital length of stay for internal medicine inpatients with cardiopulmonary diagnosis at admission: study protocol of a randomized controlled trial—the IMFCU-1 (Internal Medicine Focused Clinical Ultrasound) study
Source: Trials. 2020 Jan 8;21:53. doi: 10.1186/s13063-019-4003-2 (PMC6951003; doi:10.1186/s13063-019-4003-2)
Supplement: Supplementary file 2 — Additional file 2. Clinical Assessment. [file 13063_2019_4003_MOESM2_ESM.docx]

**Conventional Clinical Assessment**

After conducting your clinical assessment and reviewing available pathology and imaging, please complete the following diagnosis and management plans.

**BEFORE ULTRASOUND**

| Date of Assessment | DD/MM/YY |  |
| --- | --- | --- |
| Variable | Definition | Enter Value |
| Assessing Doctor Seniority | 1 = consultant, 2 = senior registrar/fellow, 3 = junior registrar |  |
| Haemodynamic evaluation |  |  |
| Haemodynamic state | 1= normal 2 = hypovolaemia 3= Primary diastolic failure 4= Primary Systolic failure  5= Systolic and Diastolic failure,  6= Vasodilation, 7=Right ventricle failure |  |
| Heart |  |  |
| Left ventricular function | 1 = normal, 2= mild/moderate reduction (EF 30-55%) 3= severe reduction (EF<30%) |  |
| Valves (Mark only moderate or severe abnormality) | |  |
| Aortic valve | 1= normal 2= regurgitation 3= stenosis |  |
| Mitral valve | 1= normal 2= regurgitation 3= stenosis |  |
| Tricuspid valve | 1= normal 2= regurgitation 3= stenosis |  |
| Pulmonary valve | 1= normal 2= regurgitation 3= stenosis |  |
| Significant pericardial effusion | 1= nil, 2 = yes |  |
| Pulmonary embolus | 1= nil, 2= yes |  |
| Lungs |  |  |
| Is there evidence of obstructive airways disease (such as wheezing) | 1 = nil, 2 = yes |  |
| Left side |  |  |
| Pleural effusion | 1= nil, 2 = yes |  |
| Interstitial/alveolar infiltrates | 1= nil, 2 = yes |  |
| Consolidation | 1= nil, 2 = yes |  |
| Collapse (atelectasis) | 1= nil, 2 = yes |  |
| Pneumothorax | 1= nil, 2 = yes |  |
| Right side |  |  |
| Pleural effusion | 1= nil, 2 = yes |  |
| Interstitial/alveolar infiltrates | 1= nil, 2 = yes |  |
| Consolidation | 1= nil, 2 = yes |  |
| Collapse (atelectasis) | 1= nil, 2 = yes |  |
| Pneumothorax | 1= nil, 2 = yes |  |
| Lower extremities |  |  |
| Is there evidence of deep venous thrombosis (DVT) | 1= nil, 2= clinical suspicion, 3= DVT confirmed by imaging |  |
| Other medical conditions |  |  |
| Document any other medical conditions that will influence your management |  |  |
| Admission diagnosis | Most likely |  |
|  | Second most likely |  |
|  | Third most likely |  |

# Management Plan –BEFORE ULTRASOUND

Please document your planned management below

| Date of management plan | | DD/MM/YY |  |
| --- | --- | --- | --- |
| Management | | Definition | Enter value |
| Investigations | |  |  |
| **Blood tests** | | 1= nil, 2 = yes |  |
| If the answer is “yes” please mark which tests from the list below | | |  |
|  | FBE |  |  |
|  | U+E |  |  |
|  | LFT |  |  |
|  | ABG |  |  |
|  | ESR |  |  |
|  | CRP |  |  |
|  | Troponin/CkMB |  |  |
|  | Pro-BNP |  |  |
|  | Urine test |  |  |
|  | Blood cultures |  |  |
|  | Sputum/tissue/urine/wound cultures |  |  |
|  | Other |  |  |
| **Imaging** | | 1= nil, 2 = yes |  |
| If the answer is “yes” please mark which imaging from the list below | | |  |
|  | Chest X ray |  |  |
|  | Chest CT scan |  |  |
|  | CT scan other than chest |  |  |
|  | MRI |  |  |
|  | Echocardiography |  |  |
|  | Other |  |  |
| **Consultation – medical specialty** | | 1= nil, 2= yes |  |
| If the answer is “yes” please mark which specialty from the list below | | |  |
|  | Cardiology |  |  |
|  | Respiratory |  |  |
|  | Nephrology |  |  |
|  | Infection disease |  |  |
|  | Critical care |  |  |
|  | Other |  |  |
| Treatment | |  |  |
| Heart failure treatment | | 1 = no, 2 = yes  Definition: includes at least one of the following treatment: diuretics, vasodilators and/or fluid restriction. |  |
| COPD/Asthma treatment | | 1 = no, 2 = yes  Definition: bronchodilation and/or systemic corticoid |  |
| Antibiotics | | 1= no, 2 = yes |  |
| Anticoagulation – therapeutic dose | | 1 = no, 2 = yes |  |
| Other | |  |  |

**AFTER ULTRASOUND**

| Date of Assessment | DD/MM/YY |  |
| --- | --- | --- |
| Variable | Definition | Enter Value |
| Assessing Doctor Seniority | 1 = consultant, 2 = senior registrar/fellow, 3 = junior registrar |  |
| Haemodynamic evaluation |  |  |
| Haemodynamic state | 1= normal 2 = hypovolaemia 3= Primary diastolic failure 4= Primary Systolic failure  5= Systolic and Diastolic failure,  6= Vasodilation, 7=Right ventricle failure |  |
| Heart |  |  |
| Left ventricular function | 1 = normal, 2= mild/moderate reduction (EF 30-55%) 3= severe reduction (EF<30%) |  |
| Valves (Mark only moderate or severe abnormality) | |  |
| Aortic valve | 1= normal 2= regurgitation 3= stenosis |  |
| Mitral valve | 1= normal 2= regurgitation 3= stenosis |  |
| Tricuspid valve | 1= normal 2= regurgitation 3= stenosis |  |
| Pulmonary valve | 1= normal 2= regurgitation 3= stenosis |  |
| Significant pericardial effusion | 1= nil, 2 = yes |  |
| Pulmonary embolus | 1= nil, 2 = yes |  |
| Lungs |  |  |
| Is there evidence of obstructive airways disease (such as wheezing) | 1 = nil, 2 = yes |  |
| Left side |  |  |
| Pleural effusion | 1= nil, 2 = yes |  |
| Interstitial/alveolar infiltrates | 1= nil, 2 = yes |  |
| Consolidation | 1= nil, 2 = yes |  |
| Collapse (atelectasis) | 1= nil, 2 = yes |  |
| Pneumothorax | 1= nil, 2 = yes |  |
| Right side |  |  |
| Pleural effusion | 1= nil, 2 = yes |  |
| Interstitial/alveolar infiltrates | 1= nil, 2 = yes |  |
| Consolidation | 1= nil, 2 = yes |  |
| Collapse (atelectasis) | 1= nil, 2 = yes |  |
| Pneumothorax | 1= nil, 2 = yes |  |
| Lower extremities |  |  |
| Is there evidence of deep venous thrombosis (DVT) | 1= nil, 2= clinical suspicion, 3= DVT confirmed by imaging |  |
| Other medical conditions |  |  |
| Document any other medical conditions that will influence your management |  |  |
| Admission diagnosis | Most likely |  |
|  | Second most likely |  |
|  | Third most likely |  |

# Management Plan – AFTER ULTRASOUND

| Date of management plan | | DD/MM/YY |  |
| --- | --- | --- | --- |
| Management | | Definition | Enter value |
| Investigations | |  |  |
| **Blood tests** | | 1= nil, 2 = yes |  |
| If the answer is “yes” please mark which tests from the list below | | |  |
|  | FBE |  |  |
|  | U+E |  |  |
|  | LFT |  |  |
|  | BG |  |  |
|  | ESR |  |  |
|  | CRP |  |  |
|  | Troponin/CkMB |  |  |
|  | Pro-BNP |  |  |
|  | Urine test |  |  |
|  | Blood cultures |  |  |
|  | Sputum/tissue/urine/wound cultures |  |  |
|  | Other |  |  |
| **Imaging** | | 1= nil, 2 = yes |  |
| If the answer is “yes” please mark which imaging from the list below | | |  |
|  | Chest X ray |  |  |
|  | Chest CT scan |  |  |
|  | CT scan other than chest |  |  |
|  | MRI |  |  |
|  | Echocardiography |  |  |
|  | Other |  |  |
| **Consultation – medical specialty** | | 1= nil, 2= yes |  |
| If the answer is “yes” please mark which specialty from the list below | | |  |
|  | Cardiology |  |  |
|  | Respiratory |  |  |
|  | Nephrology |  |  |
|  | Infection disease |  |  |
|  | Critical care |  |  |
|  | Other |  |  |
| Treatment | |  |  |
| Heart failure treatment | | 1 = no, 2 = yes  Definition: includes at least one of the following treatment: diuretics, vasodilators and/or fluid restriction. |  |
| COPD/Asthma treatment | | 1 = no, 2 = yes  Definition: bronchodilation and/or systemic corticoid |  |
| Antibiotics | | 1= no, 2 = yes |  |
| Anticoagulation – therapeutic dose | | 1 = no, 2 = yes |  |
| Other | |  |  |
